# Analysis of Chlorogenic Acid in Sweet Potato Leaf Extracts

**DOI:** 10.3390/plants11152063

**Published:** 2022-08-07

**Authors:** Chun-Hui Chiu, Kuan-Hung Lin, Hsin-Hung Lin, Wen-Xin Chu, Yung-Chang Lai, Pi-Yu Chao

**Affiliations:** 1 Graduate Institute of Health Industry and Technology, Research Center for Chinese Herbal Medicine, Research Center for Food and Cosmetic Safety, College of Human Ecology, Chang Gung University of Science and Technology, Taoyuan 33303, Taiwan; 2 Department of Traditional Chinese Medicine, Keelung Chang Gung Memorial Hospital, Keelung 20401, Taiwan; 3 Department of Horticulture and Biotechnology, Chinese Culture University, Taipei 11114, Taiwan; 4 Department of Agronomy, National Chung Hsing University, Taichung 40277, Taiwan; 5 Graduate Institute of Biotechnology, Chinese Culture University, Taipei 11114, Taiwan; 6 Agronomy Division, Chiayi Agricultural Experiment Branch, Taiwan Agricultural Research Institute (TARI), Concil of Agriculture (COA), Executive Yuan, Chiayi 60044, Taiwan; 7 Department of Nutrition and Health Sciences, Chinese Culture University, Taipei 11114, Taiwan

**Keywords:** sweet potato leaf, chlorogenic acids, liquid chromatography-tandem mass spectrometry

## Abstract

Sweet potato (*Ipomoea batatas* L.) is one of the most important food crops worldwide, with leaves of different varieties showing purple, green and yellow, and these leaves provide a dietary source of nutrients and various bioactive compounds. The objective of this study was to identify the active constituents of chlorogenic acids (CGAs) in different methanolic extract of leaves of three varieties of sweet potato (purple CYY 98-59, green Taoyuan 2, and yellow CN 1927-16) using liquid chromatography–tandem mass spectrometry. Genotype-specific metabolite variations were observed; CGAs and three isomeric peaks were detected in sweet potato leaf extracts (SPLEs). Among them, the yellow SPLE contained the highest contents of 3,5-dicaffeoylquinic acid (3,5-di-CQA) and 3,4-dicaffeoylquinic acid (3,4-di-CQA), followed by the green SPLE, whereas the purple SPLE retained lower 3,5-di-CQA content compared to yellow and green SPLEs. All three SPLEs contained lower 4,5-dicaffeoylquinic acid (4,5-di-CQA) and CGA contents compared to 3,5-di-CQA and 3,4-di-CQA, although CGA constituents were not significantly different in genotypes, whereas purple SPLE contained higher 4,5-di-CQA content compared to yellow and green SPLEs. This study indicates that SPLs marketed in Taiwan vary widely in their biological potentials and may impart different health benefits to consumers.

## 1. Introduction

There is increasing interest in using antioxidants from plants as functional foods and nutraceutical products with antioxidant properties. There is also great interest in the use of potent dietary antioxidants in preventive strategies, with applications ranging from oxidative reactions in foods and pharmaceuticals to the role of reactive oxygen species (ROS) in chronic degenerative diseases [1,2]. On a global scale, sweet potato (*Ipomoea batatas* L.) ranks fifth after the cultivation of rice, wheat, corn, and cassava [3]. Sweet potato leaves (SPLs) are a leafy vegetable consumed by humans and are currently widely used for food due to their high yield, drought tolerance, and ability to grow in different climates and farming systems [4]. SPLs contain higher amounts of sugars, proteins, minerals, and various vitamins than other leafy vegetables [5,6], and are rich in polyphenols and various other components [7]. It is necessary to select appropriate fresh SPLs for commercial-scale extraction to obtain an end product with a very high content of chlorogenic acid (CGA) derivatives.

**Figure 1:**
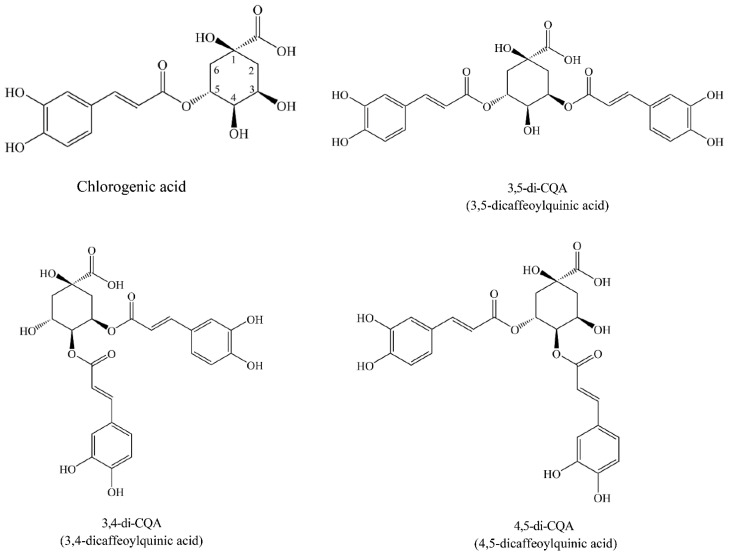
Structure of chlorogenic acid (CGA) and isochlorogenic acids, 3,5-dicaffeoylquinic acid (3,5-di-CQA), 3,4-dicaffeoylquinic acid (3,4-di-CQA), and 4,5-dicaffeoylquinic acid (4,5-di-CQA).

CGAs are among the most abundant polyphenols in the human diet, and their biologically active compounds are shown to have various health benefits and antioxidant properties [8]. CGAs are complex molecules exhibiting different physiochemical properties due to positional esterification on the quinic acid moiety, leading to regioisomers [9]. CGA consists of one or more cinnamic acids, including *p*-coumaric acid, caffeic acid (CA), and any molecule formed by the ester bond between ferulic acid (FA) and quinic acid (QA), resulting in *p*-coumarinylquinic acids (CoQA), caffeoylquinic acids (CQAs), and feruloylquinic acids (FQAs) [10,11]. The main phenolic components of SPLs are CA, QA, and derivatives such as 3,4,5-tri-CQA (3,4,5-tricaffeoylquinic acid), 4,5-di-CQA (4,5-dicaffeoylquinic acid), 3-mono-CQA, 3,4-di-CQA (3,4-dicaffeoylquinic acid), and 3,5-di-CQA (3,5-dicaffeoylquinic acid). Furthermore, isochlorogenic acid A, isochlorogenic acid B, and isochlorogenic acid C are also termed 3,5-di-CQA, 3,4-di-CQA, and 4,5-di-CQA, respectively (Figure 1) [12,13,14], and the constituents are associated with the specific genotypes [15]. The total phenolic compounds of eight Japanese SPL varieties range from 6.3 to 13.5 g GAE/100 g DW [16], while four Taiwanese varieties (TNG10, TNG57, TNG66, and YSP) have relatively lower contents due to the water extraction method used [17]. Zhang et al. [18] identified 37 compounds in SPLs with ethyl acetate extracts, including 20 phenolic acids, 12 flavonoids, 3 organic acids, 1 nucleoside, and 1 ester. Among them, caffeic acid ethyl ester, trans-N-feruloyltyramine, cis-N-feruloyltyramine, trans-N- (*p*-coumaroyl) tyramine, 4,5-feruloyl courmaoylquinic acid, 7-hydroxy-5-methoxycoumarin, 7,3′-dimethylquercetin, and indole-3-carboxaldehyde were first detected in sweet potato leaves [19]. Leaves of nine sweet potato cultivars grown in central Europe contained 7 polyphenolic compounds, including 5 CGAs (3-CQA, 4-CQA, 5-CQA, 3,4-di-CQA, and 3,5-di-CQA) and 2 flavonoids (quercetin-3-*O*-galactoside and quercetin-3-*O*-glucoside) [20]. However, bioactivity data of Taiwanese indigenous SPL on CGA derivatives have so far been only sporadically studied, and limited information is available on the CGA composition of these dietary sweet potato genotypes. Increasing CGA content in SPLs is a goal for producing food security and providing available nutrients to a large portion of the world’s population.

Previously, we illustrated that more than half of 27 indigenous Taiwanese colored vegetables contained quercetin, morin, and myricetin, and that purple SPL exhibited especially higher antioxidant activity than others due to its higher cyanidin, quercetin, and polyphenol levels compared to other vegetables [21]. Furthermore, we reported that purple SPLE and its components, cyanidin and quercetin, have anti-inflammatory effects through modulation of NFκB and decreased expression of ERK1, ERK2, and p38 MAPK on human aortic endothelial cells (HAECs) were pretreated with 100 μg/mL purple SPLE or 10 μM cyanidin and quercetin for 18 h, followed by TNF-α (2 ng/mL) for 6 h. The results from our previous study show that purple SPLE, cyanidin, and quercetin significantly inhibited TNF-α-induced monocyte-endothelial cell adhesion and attenuated ICAM-1 and E-selection expression [22]. We also found that yellow, purple, and red SPLEs can improve tumor necrosis factor (TNF)-α-induced insulin resistance by activating insulin signaling, thus resulting in increased glucose uptake [23]. The higher contents of total phenols, flavonoids, and anthocyanins, and antioxidant properties in these colored leaves may be the reason for their wide medicinal use. Some indigenous Taiwanese SPLs have unique biologically active components, polyphenols and flavonoids being the main components, that have potential health-promoting benefits including antioxidant, anti-diabetic, anti-cancer, anti-hepatotoxicity, antihypertensive, anti-inflammatory, and antibacterial effects [24,25,26,27,28,29]. However, there is still a need to identify complete profiles of CGAs related to their biological activities, especially the qualitative and quantitative analysis of the bioactive components present in the leaves of three sweet potato cultivars. The results of our study may be used to develop future SPLs for food and medicinal industries and produce high-value nutraceuticals.

## 2. Results and Discussion

### 2.1. The Extraction Yields of Different Sweet Potato Leaf

The extraction yields of purple, green, and yellow SPLEs were 24.03 ± 0.24%, 18.50 ± 0.25%, and 17.27 ± 0.37%, respectively. Accordingly, the yields of SPLE fractions showed obvious variation due to variable water content. The methanol extract of sweet potato leaves has the highest phenolic acid content, followed by the peel, whole root, and fleshy tissue [30]. In one study, the extraction rates of purple SPLE were significantly higher than green and yellow SPLEs, indicating that purple SPLE exhibited higher cyanidin, quercetin, and polyphenol levels [21].

### 2.2. LC-MS/MS Analysis of CGA and CQA

**Figure 2:**
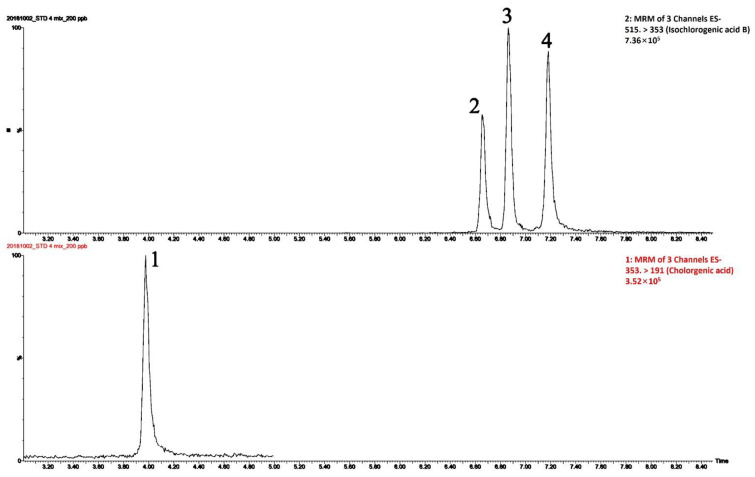
LC-MS/MS analysis of chlorogenic acid (CGA, peak 1), 3,4-dicaffeoylquinic acid (3,4-di-CQA, peak 2), 3,5-dicaffeoylquinic acid (3,5-di-CQA, peak 3), and 4,5-dicaffeoylquinic acid (4,5-di-CQA, peak 4).

CGA and CQAs were identified from their precursor ions, fragmentation patterns, and retention times, as shown in Table 1 and Figure 2. One single base peak was detected in each compound standard, and values of all peaks were relatively steadily distributed in SPLE samples. The retention time of the peaks in the mass spectra of standard CGA, 3,4-di-CQA, 3,5-di-CQA, and 4,5-di-CQA were successfully separated in 7.5 min. The signal- to-noise (s/n) ratios (18.2–30.2) of each analyte at 5 ng mL^−1^ were all greater than 10 (data not shown). In addition, the calibration curve of GCA, 3,5-di-CQA, 3,4-di-CQA, and 4,5-di-CQA compounds were analyzed from 5 to 200 ng mL^−1^, the correlation coefficients (*r*) values of which were all higher than 0.99 (Table 1) and the method showed that the results had good linearity. CGAs are chemically diverse, and their various regio- and geometrical isomers can make discrimination challenges. The elution order of these metabolites was considered to assist in their annotation, and mass spectrometric data obtained [M + H]- as the parent ion in ESI negative modes and chromatographic elution order were also considered when determining the regio- and geometric isomers of annotated metabolites. The metabolite with a precursor ion at m/z 353 was annotated as CGA, as it produced a fragment ion at m/z 191. Moreover, the identification of metabolites with the parent ion at m/z 515 was identified as CQAs, as they produced a product ion at m/z 353. 3,5-di-CQA, 3,4-di-CQA, and 4,5-di-CQA had the same parent ion (m/z 515) and product ion (m/z 353), which were detected separately based on their differential retention times. Hence, based on elution order and fragmentation patterns, metabolites identified as GCA, 3,5-di-CQA, 3,4-di-CQA, and 4,5-di-CQA were at peak 1, peak 3, peak 2, and peak 4, respectively.

### 2.3. CGA and CQAs Contents of Different Sweet Potato Leaf

**Figure 3:**
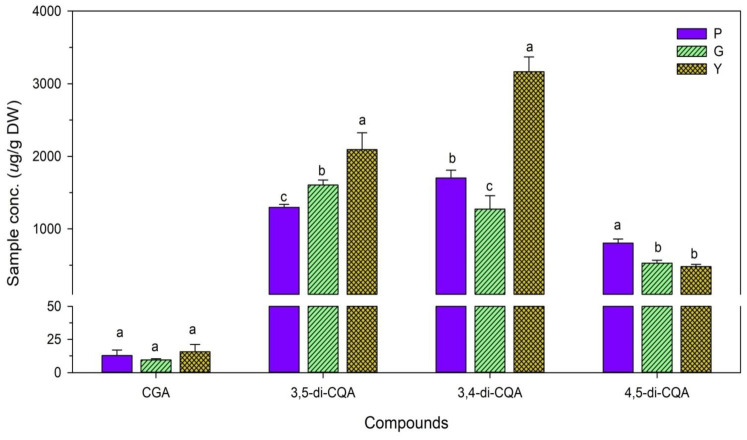
Content (μg/g DW) of chlorogenic acid (CGA), 3,5-di-CQA (3,5-dicaffeoylquinic acid), 3,4-di-CQA (3,4-dicaffeoylquinic acid), and 4,5-di-CQA (4,5-dicaffeoylquinic acid) of purple (P, CYY 98-59), green (G, Taoyuan 2), and yellow (Y, CN 1927-16) leaf extracts. All MS/MS data for analytes were collected in the multiple reaction monitoring (MRM) mode, using MassLynx v4.1 software. The concentrations of CGAs were expressed as means ± standard deviations (*n* = 3). Means with different letters among the three genotypes of sweet potato leaves indicate statistical significance at *p* < 0.05.

Mean SPL levels of the four peaks calculated as the concentrations (μg/g DW) from all SPL tested samples are shown in Figure 3. Both 3,5-di-CQA and 3,4-di-CQA were rich in the yellow SPLs at levels of 2094 ± 230 μg/g DW and 3167 ± 203 μg/g DW, respectively. All yellow SPLs had significantly higher 3,5-di-CQA and 3,4-di-CQA contents than other genotypes, suggesting that 3,5-di-CQA and 3,4-di-CQA compounds both play important roles in bioactivity in yellow SPLs. The amounts of CGA (9~15 μg/g DW) and 4,5-di-CQA (480~804 μg/g DW) compounds found in all genotypes were notably lower than the amounts of 3,5-di-CQA (1300~2100 μg/g DW) and 3,4-di-CQA (1270~3170 μg/g DW) detected. CGA in all samples did not show any significant differences among genotypes, whereas purple SPLs (804.27 ± 56.60 μg/g DW) contained more 4,5-di-CQA compared to green (527.20 ± 41.10 μg/g DW) and yellow (480.51 ± 31.80 μg/g DW) SPLs. Jung et al. [31] and Krochmal–Marczak et al. [20] reported that the dominating compounds in sweet potato leaves were 5-CQA and 3-CQA, respectively. The identification and quantification of structurally related compounds are challenging and may require reliable advanced analytical techniques. The 3,4-di-CQA contents (Figure 3) found in our study were higher than in a previous study [30], which may have been due to our methanolic extraction compared to their 80% ethanol extraction. Different extraction solvents result in differences in extract compositions, and consequently, apparent bioactivities.

Many researchers reported that the influence of different extraction solvents, such as methanol, ethanol, acetone, propanol, and ethyl acetate, have been commonly used to extract phenolics from fresh plant leaves [32,33]. In addition, the drying method also affects the contents of CGA derivatives in the SPL, with freeze-drying retaining the highest amount of total CGA derivatives, followed by 30 °C cool air drying [34]. In our study, changes in CQA contents during sample extraction may be dependent on the sweet potato genotype and solvent used, and the polarities of SPLEs differ due to extracts from the three different genotypes having different CQA compounds, which could influence their uptake and cellular distributions [35,36]. CQAs are lipophilic, which may affect the specific interaction of each compound’s hydrophobicity, and which in turn would affect its distribution within leaf tissue [37,38]. CQA compounds in SPLEs are therefore multifunctional, and their activities and mechanisms of action largely depend on the composition and conditions of the test system.

Plant secondary metabolites are important in the production of flavors, pharmaceuticals, food additives, and many other applications; therefore, ways to produce important secondary metabolites effectively at a large scale are needed [39]. In this study, SPLEs reveal their potential to be developed as active ingredients or food additives, thus increasing the economic value of Taiwanese SPLs in the food industry. Food supplements and diets containing CGAs and derivatives such as SPL, either in dry form, capsules, or tea, may be useful in reducing oxidative damage and helping the food and drug industry alleviate oxidative-induced chronic diseases. CGAs and their derivatives are normal constituents of the human diet, since they are present in colored vegetables. The latter have also been used as additives for food coloration purposes. These different CGAs and derivatives may exhibit effective biological activities and are a likely cause of differences among these cultivars. The determination of CGAs and derivatives in SPLEs is valuable for increasing the bioactivity of SPL products. Therefore, effective-dose in vivo studies are worthy of further investigation. SPLs are found abundantly in any market in Taiwan, and they impart various health benefits to consumers. SPLE can be used to develop products with high nutraceutical value, thus playing a significant role in providing good nutrition and improving human health. People can consume SPLs as a low-cost, nutritious food, and use them as a low-cost medicine for the treatment of diseases. CGA derivatives are generally abundant in yellow SPLs, so the latter could provide an accessible chemical resource for testing different genotypes to maximally harness bioactivity. The results of such research could also help consumers select yellow SPLs with high levels of health-promoting compounds. In addition, the profiling of CGA in differentiated genotypes contributes to the possible identification of any underlying biochemical mechanisms concerning CGA biosynthesis among genotypes. The over-expression of the IbPAL1 gene promotes CGA accumulation and biosynthetic pathway gene expression in the leaves of Sushu 16, providing it with nutritional quality improvement [40]. Furthermore, sweet potato breeders can also use this information to develop genotypes with superior health-benefiting properties and produce specific leaf colors preferred by consumers.

Since some of the SPL possesses good antioxidant activity, its protective effects may come from its rich content of CGA and derivatives. Surprisingly, CGA compounds occur at extremely low levels, suggesting that the amounts of CGA in all SPLEs are in very low concentrations. Alternatively, the CGA may be converted to 4,5-di-CQA, 3,4-di-CQA, or 3,5-di-CQA derivatives by removal of the ester bond between FA and QA. Meanwhile, 3-CQA and 3,5-di-CQA are stable under acidic conditions, while the isomerization of 3-CQA to 4-CQA/5-CQA and 3,5-di-CQA to 3,4-di-CQA and 4,5-di-CQA occur rapidly at neutral and basic pH values [38]. Bolanos et al. [41] demonstrated that 3,5-di-CQA was present in the highest amounts in SPLs, and its content depended on the genotype of examined cultivars and the 4,5-di-CQA stage of leaf development [19]. Differences observed in the intensities of fragment ions can be ascribed to variances in energy distribution, which cause structurally similar isomers to behave differently under the described mass spectrometric conditions. Alternatively, 3,5-di-CQA and 3,4-di-CQA derivatives might be more stable when being extracted in methanol than CGA and 4,5-di-CQA compound derivatives, which indicates several possibilities that suggest further investigation. Anti-diabetic effects have been demonstrated for several components of SPLs, such as myricetin [42], CA derivatives [43], CGA [44], anthocyanins [45], and the stage of leaf development CQA derivatives [19]. The therapeutic potential of CGAs as anti-diabetic agents for use in certain disorders and a full framework of CGA metabolic pathways in the human body are worthy of further investigation. The mechanisms and intensities of anti-diabetic effects may depend on the particular CGA derivative used. Do CGAs have specific biological functions in vivo? Currently, we are working on the CGAs of SPLEs to test whether any CGA compound can improve the intake of 2-NBDG through the expression of GLUT4 in myoblast cell line C2C12.

## 3. Materials and Methods

### 3.1. Source of SPLs and Preparation of SPLEs

Three varieties and breeding lines of purple (CYY 98-59), green (Taoyuan 2), and yellow (CN 1927-16) leaves of sweet potato (*Ipomoea batatas* L.) were provided and identified by the Department of Agronomy, Chiayi Agricultural Experiment Station, Chiayi, Taiwan. Leaves were washed, air-dried, weighed, and lyophilized (Freeze Dryer- 5060, Panchum Scientific, Taipei, Taiwan). Dried samples were then ground into powder, screened through a 40-mesh sieve (with an aperture of 0.475 mm), and stored at −80 °C. A half gram of powder from each sample was extracted with 5 mL of methanol, stirred on a stirring plate at 25 °C for 2 h, centrifuged at 4 °C and 12,000 rpm for 10 min, followed by collection of the supernatant. The remaining residue was re-extracted twice until the residue was colorless. The three extracts were combined, filtered through #1 filter paper (Whatman, Hillsboro, OR, USA), and the filtrate concentrated in vacuo at 25 °C to dryness to obtain the methanolic extract. Dried filtrates were weighed to determine the extracted yield of soluble constituents. The extraction yield (%) was calculated as the dried filtrate weight (g) divided by the sample weight (g, dry mass) × 100%. Methanolic extracts were then stored at −80 °C for further assays.

### 3.2. Ultra Performance Liquid Chromatography-Triple Quadrupole Mass Spectrometry (UPLC-MS/MS)

One hundred micrograms of SPLE powder were dissolved in 100% methanol and then made up to 5 mL as an SPLE solution. Solid-phase extraction cartridges (streata^TM^ 33 μm, 200 mg/3 mL, Phenomenex, Denver, CO, USA) were conditioned with 6 mL methanol and 6 mL ultrapure water (Direct-Q^®^ 3, Merck, Darmstadt, Germany) before being loaded with SPLE samples. A 0.5 mL SPLE solution was injected into the activated solid-phase extraction cartridges and then washed with 6 mL of 10% methanol. Elution was carried out with 6 mL 100% methanol and added to a final volume of 10 mL with 100% methanol. The fraction was then filtered using a 0.2 μm filter (PureTech, 13 mm Nylon, Taiwan) into a UPLC glass vial.

UPLC-ESI-MS/MS analysis was performed using a Waters Acquity Ultra Performance LC system (Waters, Milford, MA, USA) equipped with a TQS triple quadrupole MS/MS system (Waters, Milford, MA, USA). The Acquity UPLC BEH C8 column (150 mm × 2.1 mm with particle size of 1.7 μm) was maintained at 30 °C at a flow rate of 0.3 mL/min. A binary solvent system was used that consisted of 5% acetonitrile with 0.1% formic acid (solvent A) and 100% acetonitrile with 0.1% formic acid (solvent B). The gradient elution program was performed as follows: 0–1 min, 5% B; 1–10 min, 41% B; 10–11 min, 99% B; 11–12 min, 99% B; 12–13 min, 5% B; and 13–15 min, 5% B. The injection volume was 2 µL via autosampler. Separate injections (using the same chromatographic settings and conditions) were performed for negative electrospray ionization (ESI) modes. ESI parameters were as follows: capillary voltage of 2.5 kV, sampling cone voltage of 2 V, desolvation temperature of 200 °C, cone gas flow of 150 L/h, and desolvation gas flow of 800 L/h. Data were acquired and processed using Mass Lynx 4.1 software (Waters, Milford, MA). To ensure experimental reproducibility, three independent biological replicates were prepared, and three instrumental technical replicates were analyzed.

Standards for CGA, chlorogenic acid (CGA), 3,5-dicaffeoylquinic acid (3,5-di-CQA or isochlorogenic acid A), 3,4-dicaffeoylquinic acid (3,4-di-CQA or isochlorogenic acid B), and 4,5-dicaffeoylquinic acid (4,5-di-CQA or isochlorogenic acid C) were purchased from Sigma-Aldrich (Missouri, USA). For content measurement of CGA and three ICGAs in SPLE, standard solutions in a concentration range of 5 to 200 ng mL^−1^ containing CGA, 3,5-di-CQA, 3,4-di-CQA, and 4,5-di-CQA were freshly prepared in 50% methanol.

### 3.3. Identification and Determination of CGAs and ICGAs by UPLC-MS/MS

Quantification of CGAs and ICGAs were performed using MRM on a UPLC-MS/MS system. The concentrations of CGAs or ICGAs in SPL samples (ug/g DW) were calculated using the following equation: (C × 20 × V × F)/M × 1000, where C was the concentration of each compound in the test solution from the standard curve (ng mL^−1^), V was the final volume of the sample (mL), M was the amount of sample in grams, and F was the dilution factor of the test solution.

### 3.4. Statistical Analysis

All analyses were determined in triplicate, and results are expressed as means and standard deviations (SDs). An analysis of variance (ANOVA) with the Duncan’s test at 0.05 was performed using SPSS version 23.0 (SPSS, Chicago, IL, USA).

## 4. Conclusions

Taiwanese SPLs were identified as containing potent polar antioxidants, confirming that 3,5-di-CQA and 3,4-di-CQA account for high proportions of CQAs and are the main isomers of CQAs in SPLs. SPLEs from different genotypes displayed variations in antioxidant substances. These compounds occur in relatively major amounts in the yellow SPLE, and have been shown to have large bioactivity, which is likely to impact human health. SPLs can be viable and economical sources of antioxidants in the diet. Some of the beneficial effects of CGA derivates might be mediated through their effects on metabolic pathways and biological functions for possible clinical applications. Our observations may enhance the potential application of SPLs as an inexpensive source of natural antioxidants, especially for the currently fast-growing functional food industry.

## Figures and Tables

**Table 1:** Retention time (RT), parent ions, product ions, linear equation (5~200 ng mL^−1^), and correlation coefficient (*r*) of each compound standard.

Compound	ESI Mode	RT (min)	Parent Ions (m/z)	Product Ions (m/z)	Linear Equation	*r*
Chlorogenic acid	-	3.98	353	191	Y = 7.41X − 30.9	0.9973
3,5-di-CQA	-	6.86	515	353	Y = 100.83X − 298.9	0.9976
3,4-di-CQA	-	6.65	515	353	Y = 66.52X − 357.6	0.9989
4,5-di-CQA	-	7.17	515	353	Y = 99.79X − 350.5	0.9997

## Data Availability

Not applicable.

## References

[1] Bayr H. (2005). Reactive Oxygen Species. Crit. Care Med..

[2] Brewer M. (2011). Natural antioxidants: Sources, compounds, mechanisms of action, and potential applications. Compr. Rev. Food Sci. Food Saf..

[3] Food and Agriculture Organization of the United Nations (2020). FAO STAT Statistics Database.

[4] Lin K.H., Chao P.Y., Yang C.M., Cheng W.C., Lo H.F., Chang T.R. (2006). The effects of flooding and drought stresses on the antioxidant constituents in sweet potato leaves. Bot. Stud..

[5] Sun H., Mu T., Xi L., Zhang M., Chen J. (2014). Sweet potato (*Ipomoea batatas* L.) leaves as nutritional and functional foods. Food Chem..

[6] El Sheikha A.F., Ray R.C. (2017). Potential impacts of bioprocessing of sweet potato. Crit. Rev. Food Sci. Nutr..

[7] Fu Z.F., Tu Z.C., Zhang L., Wang H., Wen Q.H., Huang T. (2016). Antioxidant activities and polyphenols of sweet potato (*Ipomoea batatas* L.) leaves extracted with solvents of various polarities. Food Biosci..

[8] Liang N., Kitts D.D. (2016). Role of chlorogenic acids in controlling oxidative and inflammatory stress conditions. Nutrients.

[9] Ramabulana A.T., Steenkamp P., Madala N., Dubery I.A. (2020). Profiling of chlorogenic acids from *Bidens pilosa* and differentiation of closely related positional isomers with the aid of UHPLC-QTOF-MS/MS-based in-source collision-induced dissociation. Metabolites.

[10] Madala N., Tugizimana F., Steenkamp P. (2014). Development and optimization of an UPLC-QTOF-MS/MS method based on an in-source collision induced dissociation approach for comprehensive discrimination of chlorogenic acids isomers from Momordica plant species. J. Anal. Methods Chem..

[11] Clifford M.N., Jaganath I.B., Ludwig I.A., Crozier A. (2017). Chlorogenic acids and the acyl-quinic acids: Discovery, biosynthesis, bioavailability and bioactivity. Nat. Prod. Rep..

[12] Luo C., Wang X., Gao G., Wang L., Li Y., Sun C. (2013). Identification and quantification of free, conjugate and total phenolic compounds in leaves of 20 sweet potato cultivars by HPLC–DAD and HPLC–ESI–MS/MS. Food Chem..

[13] Zheng X., Renslow R.S., Makola M.M., Webb I.K., Deng L., Thomas D.G., Govind N., Ibrahim Y.M., Kabanda M.M., Dubery I.A. (2017). Structural elucidation of *cis/trans* dicaffeoylquinic acid photoisomerization using ion mobility spectrometry-mass spectrometry. J. Phys. Chem. Lett..

[14] Wianowska D., Gil M. (2019). Recent advances in extraction and analysis procedures of natural chlorogenic acids. Phytochem. Rev..

[15] Islam M.S., Yoshimoto M., Yamakawa O. (2003). Distribution and physiological functions of caffeoylquinic acid derivatives in leaves of sweet potato genotypes. J. Food Sci..

[16] Nagai M., Tani M., Kishimoto Y., Iizuka M., Saita E., Toyozaki M., Kamiya T., Ikeguchi M., Kondo K. (2011). Sweet potato (*Ipomoea batatas* L.) leaves suppressed oxidation of low density lipoprotein (LDL) in vitro and in human subjects. J. Clin. Biochem. Nutr..

[17] Liao W.C., Lai Y.C., Yuan M.C., Hsu Y.L., Chan C.F. (2011). Antioxidative activity of water extract of sweet potato leaves in Taiwan. Food Chem..

[18] Zhang L., Tu Z.C., Wang H., Fu Z.F., Wen Q.H., Chang H.X., Huang X.Q. (2015). Comparison of different methods for extracting polyphenols from *Ipomoea batatas* leaves, and identification of antioxidant constituents by HPLC-QTOF-MS^2^. Food Res. Int..

[19] Zhang L., Tu Z.C., Yuan T., Wang H., Xie X., Fu Z.F. (2016). Antioxidants and α-glucosidase inhibitors from *Ipomoea batatas* leaves identified by bioassay-guided approach and structure-activity relationships. Food Chem..

[20] Krochmal-Marczak B., Cebulak T., Kapusta I., Oszmiański J., Kaszuba J., Żurek N. (2020). The content of phenolic acids and flavonols in the leaves of nine varieties of sweet potatoes (*Ipomoea batatas* L.) depending on their development, grown in Central Europe. Molecules.

[21] Chao P.Y., Lin S.Y., Lin K.H., Liu Y.F., Hsu J.I., Yang C.M., Lai J.Y. (2014). Antioxidant activity in extracts of 27 indigenous Taiwanese vegetables. Nutrients.

[22] Chao P.Y., Huang Y.P., Hsieh W.B. (2013). Inhibitive effect of purple sweet potato leaf extract and its components on cell adhesion and inflammatory response in human aortic endothelial cells. Cell Adh. Migr..

[23] Lin K.H., Low P.Y., Chao P.Y., Shih M.C., Chiang M.C., Lai Y.C., Wu S.B. (2017). Antioxidant properties and glucose uptake effect of ethanol extracts from different sweet potato leaves prepared by lyophilization and oven-drying at 40 °C. Curr. Nutr. Food Sci..

[24] Lee C.L., Lee S.L., Chen C.J., Chen H.C., Kao M.C., Liu C.H., Chen J.Y., Lai Y.T., Wu Y.C. (2016). Characterization of secondary metabolites from purple *Ipomoea batatas* leaves and their effects on glucose uptake. Molecules.

[25] Wang S., Nie S., Zhu F. (2016). Chemical constituents and health effects of sweet potato. Food Res. Int..

[26] Zhang C., Liu D., Wu L., Zhang J., Li X., Wu W. (2019). Chemical characterization and antioxidant properties of ethanolic extract and its fractions from sweet potato (*Ipomoea batatas* L.) leaves. Foods.

[27] Nguyen H.C., Chen C.C., Lin K.H., Chao P.Y., Lin H.H., Huang M.Y. (2021). Bioactive compounds, antioxidants, and health benefits of sweet potato leaves. Molecules.

[28] Bongiorno D., DiStefano V., Indelicato S., Avellone G., Ceraulo L. (2021). Bio-phenols determination in olive oils: Recent mass spectrometry approaches. Mass Spectrom. Rev..

[29] Bernhoft A. (2010). A brief review on bioactive compounds in plants. In: Bioactive compounds in plants-benefits and risks for man and animals. Nor. Acad. Sci. Lett..

[30] Truong V.D., McFeeters R., Thompson R., Dean L., Shofran B. (2007). Phenolic acid content and composition in leaves and roots of common commercial sweet potato (*Ipomea batatas* L.) cultivars in the United States. J. Food Sci..

[31] Jung J.K., Lee S.U., Kozukue N., Levin C.E., Friedman M. (2011). Distribution of phenolic compounds and antioxidative activities in parts of sweet potato (*Ipomoea batata* L.) plants and in home processed roots. J. Food Compost. Anal..

[32] Alothman M., Bhat R., Karim A. (2009). Antioxidant capacity and phenolic content of selected tropical fruits from Malaysia, extracted with different solvents. Food Chem..

[33] Lezoul N.E.H., Belkadi M., Habibi F., Guillén F. (2020). Extraction processes with several solvents on total bioactive compounds in different organs of three medicinal plants. Molecules.

[34] Jeng T.L., Lai C.C., Liao T.C., Lin S.Y., Sung J.M. (2015). Effects of drying on caffeoylquinic acid derivative content and antioxidant capacity of sweet potato leaves. J. Food Drug Anal..

[35] Miranda-Vilela A.L., Resck I.S., Grisolia C.K. (2008). Antigenotoxic activity and antioxidant properties of organic and aqueous extracts of pequi fruit (*Caryocar brasiliense* Camb.) pulp. Genet. Mol. Res..

[36] Tomsone L., Kruma Z., Galoburda R. (2012). Comparison of different solvents and extraction methods for isolation of phenolic compounds from horseradish roots (*Armoracia rusticana*). Intern. J. Agric. Biosyst. Eng..

[37] Clifford M.N., Wu W., Kirkpatrick J., Kuhnert N. (2007). Profiling the chlorogenic acids and other caffeic acid derivatives of herbal Chrysanthemum by LC− MS^n^. J. Agric. Food Chem..

[38] Xue M., Shi H., Zhang J., Liu Q.-Q., Guan J., Zhang J.-Y., Ma Q. (2016). Stability and degradation of caffeoylquinic acids under different storage conditions studied by high-performance liquid chromatography with photo diode array detection and high-performance liquid chromatography with electrospray ionization collision-induced dissociation tandem mass spectrometry. Molecules.

[39] Tiwari R., Rana C. (2015). Plant secondary metabolites: A review. Intern. J. Eng. Res. Gen. Sci..

[40] Yu Y., Wang Y., Yu Y., Ma P., Jia Z., Guo X., Xie Y., Bian X. (2021). Over expression of IbPAL1 promotes chlorogenic acid biosynthesis in sweet potato. Crop J..

[41] Bolanos J., Lee S.O., Howard L., Brownmiller C., Islam S., Rabbani M.B. (2020). Variety and Year Impact on Phenolic content of Arkansas-grown sweet potato leaves. Curr. Develop. Nutr..

[42] Knekt P., Kumpulainen J., Järvinen R., Rissanen H., Heliövaara M., Reunanen A., Hakulinen T., Aromaa A. (2002). Flavonoid intake and risk of chronic diseases. Am. J. Clin. Nutr..

[43] Jung U.J., Lee M.K., Park Y.B., Jeon S.M., Choi M.S. (2006). Antihyperglycemic and antioxidant properties of caffeic acid in db/db mice. J. Pharmacol. Exp. Ther..

[44] Bassoli B.K., Cassolla P., Borba-Murad G.R., Constantin J., Salgueiro-Pagadigorria C.L., Bazotte R.B., da Silva R.S.d.S.F., de Souza H.M. (2008). Chlorogenic acid reduces the plasma glucose peak in the oral glucose tolerance test: Effects on hepatic glucose release and glycaemia. Cell Biochem. Funct..

[45] Nagamine R., Ueno S., Tsubata M., Yamaguchi K., Takagaki K., Hira T., Hara H., Tsuda T. (2014). Dietary sweet potato (*Ipomoea batatas* L.) leaf extract attenuates hyperglycaemia by enhancing the secretion of glucagon-like peptide-1 (GLP-1). Food Funct..

